# Sarcome botryoïde du col utérin, une localisation exceptionnelle: à propos d’un cas

**DOI:** 10.11604/pamj.2023.44.102.37239

**Published:** 2023-02-22

**Authors:** Mohamed Moukhlissi, Soumya Samba, Younesse Najioui, Amal Bennani, Soufiane Berhili, Loubna Mezouar

**Affiliations:** 1 Département de Radiothérapie, Centre Régional d’Oncologie, CHU Mohammed VI, Faculté de Médecine et de Pharmacie, Université Mohamed Premier Oujda, Oujda, Maroc,; 2 Département d’Anatomie Pathologique, CHU Mohammed VI, Université Mohamed Premier Oujda, Oudja, Maroc

**Keywords:** Rhabdomyosarcome, botryoïde, col utérin, cas clinique, Rhabdomyosarcoma, botryoid, uterine cervix, case report

## Abstract

Le sarcome botryoïde est une variante de rhabdomyosarcome qui touche les tissus mous dont la localisation au niveau du col utérin reste exceptionnelle, nous rapportons l´observation d´une patiente âgée de 18 ans qui consulte aux urgences pour une pesanteur pelvienne avec des métrorragies et une rétention urinaire. L´examen gynécologique a révélé une masse bourgeonnante du col utérin dont la biopsie a montré une localisation cervicale d´un sarcome botryoïde, le bilan radiologique a montré une masse corporéo-isthmique hétérodense de 97/87 mm, sans adénopathies ni épanchements ni localisations tumorales à distance. La prise en charge a consisté à une chimiothérapie néoadjuvante à base de vincristine - Adriamycine et cyclophosphamide (V-A-C), puis une chirurgie (hystérectomie totale sans conservation annexielle). Après un suivi de 3 ans, la patiente est toujours en rémission clinique et radiologique.

## Abstract (en)

Botryoid sarcoma is a subtype of rhabdomyosarcoma affecting soft tissues and exceptionally the cervix. We here report the case of an 18-year-old female patient presenting to the emergency department with a feeling of pelvic heaviness, metrorrhagia and urinary retention. Gynecological examination showed budding mass of the uterine cervix. The biopsy showed botryoid sarcoma. Radiological evaluation revealed heterodense cervico-isthmic corporeal mass measuring 97 / 87 mm, without adenopathies or effusions or tumors at other sites. Treatment involved neoadjuvant chemotherapy with vincristine - adriamycin and cyclophosphamide (V-A-C), followed by surgery (total hysterectomy without adnexal preservation). After a follow-up of 3 years, the patient is still in clinical and radiological remission.

## Introduction

Le rhabdomyosarcome est la tumeur des tissus mous la plus courante chez les enfants, se produisant fréquemment dans le canal génital et occasionnellement dans la tête et le cou. Gonzalez-Crussi et Black-Schaffer ont classé le rhabdomyosarcome comme alvéolaire, embryonnaire, botryoïde, pléomorphique et mixte. La variante botryoïde est considérée comme un type de cellule embryonnaire apparaissant sous une muqueuse et produisant l´aspect polypoïde typique. Le sarcome botryoïde du vagin est extrêmement rare. Le sarcome botryoïde du col de l´utérus est particulièrement rare et peu de cas ont été signalés. La tumeur est généralement présente dans le col de l´utérus pendant les années de reproduction et dans le corpus utérin pendant la période postménopausique [1,2]. Sa prise en charge a été améliorée grâce à la combinaison de trois modalités thérapeutiques, la chirurgie, la chimiothérapie et la radiothérapie en fonction du degré de l´extension de la maladie [1-3]. Dans cet article, nous signalons un cas de sarcome botryoïde du col utérin chez une jeune fille de 18 ans.

## Patient et observation

**Informations relatives à la patiente:** il s´agit d´une patiente âgée de 18 ans, célibataire, ménarche à l´âge de 15 ans, étudiante et sans antécédents pathologiques particuliers, qui consulte pour des douleurs pelviennes isolées, ce qui l´a poussée à consulter chez un médecin généraliste où une échographie abdomino-pelvienne a été faite et qui a objectivé un kyste ovarien d´allure fonctionnel traité symptomatiquement. Deux mois plus tard, la patiente reconsulte aux urgences pour une pesanteur pelvienne avec des métrorragies et une rétention urinaire.

**Résultats cliniques:** l´examen gynécologique a révélé une masse bourgeonnante friable occupant la quasi-totalité du vagin empêchant de visualiser l´état du col utérin, avec perception au toucher rectal d´une masse centro-pelvienne de 10 cm.

**Figure 1:**
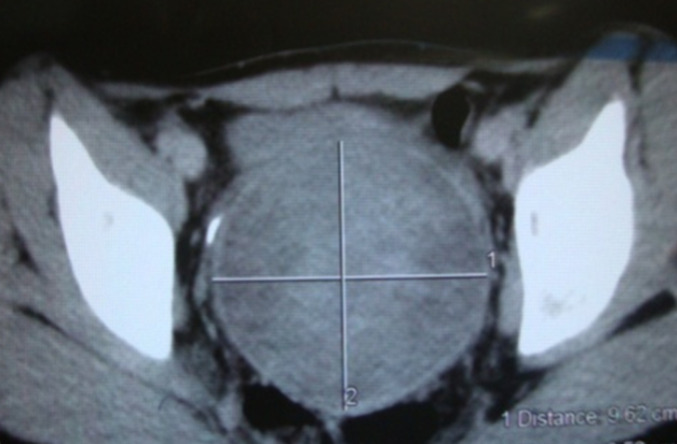
coupe de TDM abdomino-pelvienne montrant une masse corporéo-isthmique hétérodense de 97 / 87 mm, sans adénopathies ni épanchements

**Démarche diagnostique:** après sondage vésicale et traitement de la douleur une tomodensitométrie (TDM) abdomino-pelvienne a été faite en urgence et a montré une masse corporéo-isthmique hétérodense de 97 / 87 mm, sans adénopathies ni épanchements, le foie et les reins sont d´aspect normal (Figure 1).

**Figure 2:**
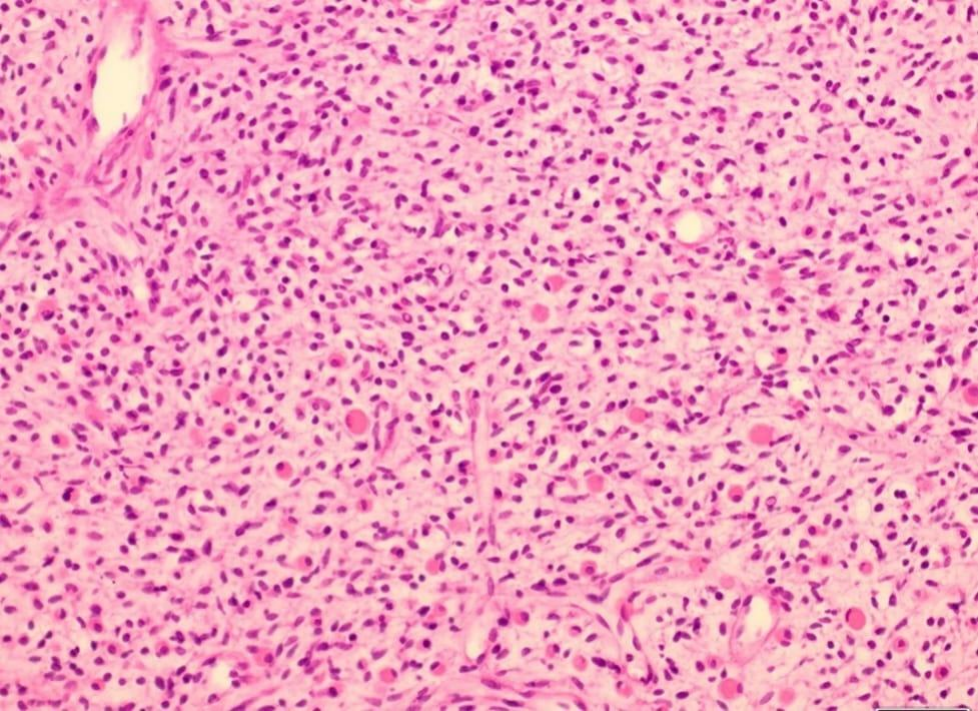
un aspect histologique d´un rhabdomyosarcome (RMS) de type embryonnaire

Des prélèvements biopsiques de la tumeur ont permis de montrer un aspect histologique d´un rhabdomyosarcome (RMS) de type embryonnaire (Figure 2). L´étude immunohistochimique montre une positivité des cellules tumorales aux anticorps dirigés contre la myogénine et la desmine et une négativité de la cytokératine confirmant le diagnostic précité. Le bilan d´extension comportant un scanner thoracique, une échographie abdominale, une cystoscopie et une rectoscopie qui étaient sans anomalies notables.

**Intervention thérapeutique:** une chimiothérapie première a été indiquée, à base de vincristine, adriamycine et cyclophosphamide (V-A-C), puis une chirurgie (hystérectomie totale sans conservation annexielle), l´étude de la pièce opératoire a montré une bonne réponse à la chimiothérapie avec des marges saines. Les suites opératoires ont été sans particularités. Le dossier a été discuté à la réunion de concertation pluridisciplinaire qui a préconisé une surveillance clinique et radiologique sans indication d´un traitement adjuvant.

**Suivi et résultats des interventions thérapeutiques:** après un suivi de 3 ans, la patiente est toujours en rémission clinique et radiologique.

**Consentement du patient:** la patiente a été informée des procédures de publication de ce cas clinique et a donné son consentement.

## Discussion

Le RMS est considéré comme une tumeur de l´enfant qui touche les tissus mous. Les localisations les plus fréquentes de ce type sont la tête, le cou, le tractus urogénital puis les extrémités [1]. On en distingue 4 types embryonnaire, alvéolaire, polymorphe et indifférencié [4]: le sarcome de type « botryoïde » est une variante du RMS embryonnaire dont la localisation utérine est extrêmement rare. Il s´agit d´une lésion sous muqueuse donnant un aspect en grappes [5], il survient en général durant la deuxième décade (entre 2 et 45 ans) [1,5,6].

Les signes cliniques ne sont pas spécifiques, le symptôme le plus souvent révélateur est un saignement vaginal anormal [1]. L´aspect macroscopique le plus évocateur est celui décrit comme une masse multivésiculaire d´aspect « molaire » [7]. La confirmation diagnostique et histologique est retenue après une étude immunohistochimique du matériel de la biopsie ou la pièce opératoire. Le sarcome botryoïde est habituellement signalé comme une tumeur vaginale dans l´appareil génital féminin des nourrissons. Cependant, il survient aussi rarement dans le col de l´utérus ou le fond de l´utérus. La survie est plus élevée et le pronostic est meilleur dans les lésions vaginales. Les taux de survie des lésions vaginales et cervicales ont été rapportés à 96% et 60% respectivement [2,6].

Les cliniciens doivent prendre soin d´examiner les lésions et les polypes du col à tout âge et toute lésion doit être biopsie. Les bienfaits de la chimiothérapie ne sont pas décrits de façon générale. Par conséquent, le patient devrait être sous étroite supervision. Quant à la prise en charge thérapeutique dont l´indication varie en fonction de l´extension de la maladie, elle regroupe la chirurgie qui constitue le traitement de base de ce genre de tumeur agressive, la chimiothérapie en particulier la chimiothérapie néoadjuvante qui permet d´augmenter le taux de la résecabilité et obtenir une chirurgie complète avec des marges saines permettant ainsi d´améliorer le pronostic et la radiothérapie est indiquée en cas de résidu tumoral ou d'adénopathie pelvienne sans preuve scientifique solide sur son efficacité [6,8].

Le pronostic du sarcome botryoïdien du col de l´utérus est plus satisfaisant que les autres rhabdomyosarcomes de l´appareil génital. L´amélioration de ce pronostic est constatée surtout lorsqu´il se présente sous forme de lésion polypoide unique et qu´il est complétement enlevé au cours de la chirurgie et reste à dire que le diagnostic précoce et le meilleur moyen pour améliorer le pronostic [2,3,6].

## Conclusion

Le sarcome botryoïde est une variante du rhabdomyosarcome, dont la localisation cervicale utérine est rare, son extension est surtout locorégionale, la chirurgie reste le traitement curatif de référence, son pronostic a été amélioré par l´introduction de la chimiothérapie et la radiothérapie.
